# COVID-19-Related Intracerebral Hemorrhage

**DOI:** 10.3389/fnagi.2020.600172

**Published:** 2020-10-22

**Authors:** Valentin Pavlov, Ozal Beylerli, Ilgiz Gareev, Luis Fernando Torres Solis, Arturo Solís Herrera, Gjumrakch Aliev

**Affiliations:** ^1^Central Research Laboratory, Bashkir State Medical University, Ufa, Russia; ^2^Department of Urology, Bashkir State Medical University, Ufa, Russia; ^3^The School of Medicine, Universidad Autónoma de Aguascalientes, Aguascalientes, Mexico; ^4^Human Photosynthesis^©^ Research Centre, Aguascalientes, Mexico; ^5^Sechenov First Moscow State Medical University, Sechenov University, Moscow, Russia; ^6^Research Institute of Human Morphology, Russian Academy of Medical Sciences, Moscow, Russia; ^7^Institute of Physiologically Active Compounds, Russian Academy of Sciences, Chernogolovka, Russia; ^8^GALLY International Biomedical Research Institute, San Antonio, TX, United States

**Keywords:** COVID-19, intracerebral hemorrhage, pathophysiological mechanisms, neurological consequences, complications

## Abstract

Intracerebral hemorrhage (ICH) is a common and severe neurological disorder and is associated with high rates of mortality and morbidity. ICH is associated with old age and underlying conditions such as hypertension and diabetes mellitus. The COVID-19 pandemic is associated with neurological symptoms and complications including ICH. For instance, the mechanisms by which COVID-19 may contribute to hemorrhagic stroke may include both depletion of angiotensin converting enzyme 2 (ACE2) receptor and overactive immune response. In this study, we herein report three patients (0.25%) out of 1200 admissions with COVID-19 to our center between 1 May and August 4, 2020, who developed ICH. In addition, we will briefly discuss the possible pathophysiological mechanisms of COVID-19 infection in patients with ICH.

## Introduction

Coronavirus infection 2019 (COVID-19) is a dangerous infectious disease that occurs as an acute respiratory viral infection with specific complications, which may include pneumonia, which leads to acute respiratory distress syndrome or respiratory failure with a high risk of death. Among the main complications of COVID-19 on the nervous system are encephalopathy, encephalitis, acute disseminated encephalomyelitis, meningitis, ischemic stroke, and intracerebral hemorrhage (ICH) and other diseases (Kannan et al., 2020). A growing number of case reports and series have been published describing the clinical characteristics of patients with ICH and COVID-19 (Table 1). The clinical course of COVID-19 is most severe in the elderly, and in patients with concomitant diseases such as hypertension and diabetes mellitus (DM), which are known to be the main factors in the development of ICH (Divani et al., 2020). Stroke has been reported as a complication of COVID-19. Most strokes among COVID-19 patients are arterial ischemic, though ICH has also been reported. We present three cases of ICH among patients hospitalized with COVID-19 from 1 May to August 4, 2020. ICH represented an infrequent complication among patients hospitalized with COVID-19 at our center, and patients with COVID-19 and ICH had other risk factors for ICH. However, our patients along with others in the literature suggest that several mechanisms may contribute to ICH in the setting of COVID-19.

**TABLE 1:** Summary of published cases of COVID-19-related ICH.

Study	Total COVID-19, n	Patients with ICH, n	Vascular risk factors	Mortality rate,%
Reddy et al., 2020	47	1	Hypertension	0%
Li et al., 2020	221	1	Not reported	100%
Morassi et al., 2020	6	2	Hypertension and thrombocytosis	100%
Al-olama et al., 2020	1	1	Meningoencephalitis	100%
Bao et al., 2020	1	1	None	0%
Benger et al., 2020	5	5	Hypertension, IHD, T2DM, and anticoagulant therapy	0%
Sharifi-Razavi et al., 2020	1	1	Not reported	Not Reported
Haddadi and Shafizad, 2020	1	1	Hypertension and diabetes	0%
Khattar et al., 2020	1	1	Hypertension	100%
Kvernland et al., 2020	4071	16	Hypertension, and anticoagulant therapy	63%
Daci et al., 2020	1	1	None	100%
Poyiadji et al., 2020	1	1	Not reported	Not Reported
Hernández-Fernández et al., 2020	1683	5	Hypertension, dyslipidemia, and T2DM	20%
Shahjouei et al., 2020	26,175 (156 stroke patients)	25	Hypertension, IHD, and T2DM	Not Reported
Ghani et al., 2020	3	3	Hypertension, anticoagulant therapy and T2DM	100%
Al-Dalahmah et al., 2020	1	1	Not Reported	100%
Gogia et al., 2020	1	1	Hypertension and dyslipidemia	100%
Nawabi et al., 2020	18	6	Hypertension and diabetes	Not Reported
Kim et al., 2020	1	1	Hypertension	0%
Dogra et al., 2020	755	33	Hypertension, dyslipidemia, T2DM, and anticoagulant therapy	42%

*ICH, intracerebral hemorrhage; IHD, Ischemic heart disease; T2DM, Type 2 diabetes mellitus.*

## Materials and Methods

### Ethics Approval and Consent to Participate

The study protocol was approved by the Hospital COVID-19 Clinics BSMU in Ufa, Russia. (Within the department of Neurosurgery), Clinic of the Bashkir State Medical University, Ufa, Republic of Bashkortostan, Russia. All research was performed in accordance with Bashkir State Medical University guidelines and regulations, and the respective authors declare a statement confirming that informed consent was obtained from all of the participants’ parents and/or their legal guardians. In addition to the guidelines described above, the authors of these manuscripts dealing with human transplantation research attesting that no organs/tissues were procured from prisoners.

### Human and Animal Rights

No animals were used for studies that are base of this research. All the humans used were in accordance with the ethical standards of the committee responsible for human experimentation (institutional and national), and with the Helsinki Declaration of 1975, as revised in 2013^1^.

We performed a retrospective chart review of all hospitalized cases with confirmed COVID-19 infection and ICH between May 1 and August 4, 2020 seen at Hospital COVID-19 Clinics BSMU in Ufa, Russia. Diagnosis of ICH was confirmed on neuroimaging with computed tomography (CT) of the brain. COVID-19 infection nucleic acid tests were performed on nasopharyngeal swabs using quantitative real-time polymerase chain reaction (qRT-PCR). Patients were included in the case series if they had tested positive for COVID-19 prior to their ICH and had continuing clinical features related to COVID-19. Inclusion criteria were defined as patients with acute ICH on CT neuroimaging and additional radiological assessment of the chest who were positive for COVID-19 and suffered from acute neurological symptoms during a hospital stay. Each of the scans had an electronic clinical and, if applicable, pathology report associated with it. Electronic reports were reviewed to extract clinical, laboratory, pathology, and demographic data. Patients were excluded if they had a secondary ICH from the hemorrhagic transformation of ischemic infarction, brain tumor, cerebral aneurysm, or vascular malformation. Baseline patient characteristics were retrieved from medical records, including symptom onset, Glasgow Coma Scale (GCS), and modified Rankin Scale (mRS) at last medical evaluation or at discharge. Additionally, vascular risk factors (hypertension, dyslipidemia and DM), laboratory parameters (C-reactive protein, D-dimer, etc.), and invasive procedures such as craniotomy from patients’ clinical records and follow-up CT were obtained. Any missing or uncertain records were collected and clarified through direct communication with health care clinicians.

## Results

A total of 1200 patients with COVID-19 were hospitalized during the 65-day study; of these, we identified three patients (0.25%) who presented with radiographic evidence of ICH and qRT-PCR-confirmed COVID-19 infection. We describe their clinical characteristics, laboratory data, imaging findings, and clinical course (Table 2).

**TABLE 2:** Baseline characteristics of patients COVID-19 with new onset of ICH during infection.

Characteristic	Patient 1	Patient 2	Patient 3
Age (years)	56	64	60
Sex	Male	Male	Male
Smoking history	Yes	Yes	No
Blood pressure (mm Hg) (at the time of ICH detection)	160/80	210/90	165/89
Blood glucose (4.0–5.4 mmol/L)	6.0	11.0	16.8
Red blood cells count (4.5–5.7 × 10^12^/L)	3.8	3.0	2.8
Hemoglobin (115–160 g/L)	96	68	75
Erythrocyte Sedimentation Rate (ESR) (<20 mm/h)	18	27	21
White blood cells count (4.0–11.0 × 10^9^/L)	14.8	15.0	15.2
Neutrophils (1.8–7.5 × 10^9^/L)	12.5	12.3	14.8
Lymphocytes (1.5–4.0 × 10^9^/L)	1.8	1.6	3.8
Eosinophils (0.0–0.4 × 10^9^/L)	0.0	0.0	0.1
Monocytes (0.2–0.8 × 10^9^/L)	1.2	0.6	0.5
Basophils (0.0–0.15 × 109/L)	0.0	0.1	0.0
Platelet count (150–450 × 109/L)	220	334	480
Creatinine (45–120 mmol/L)	66	138	130
Bilirubin, total (3–20 umol/L)	18	12	37
Total cholesterol (<5.2 mmol/L)	4.8	3.5	8.2
High-density lipoprotein (HDL) (>1 mmol/L)	1.3	1.1	1.8
Low-density lipoprotein (LDL) (<3.4 mmol/L)	2.8	3.5	5.17
D-Dimer (<500 ng/mL)	1820	2580	4000
Fibrinogen (2.0 to 4.0 g/L)	2.8	3.6	6.5
Activated partial- thromboplastin time (aPTT) (20–35 s)	22	25	21
International Normalized Ratio (INR) (0.85–1,15 ratio)	1.0	1.1	1.1
C-reactive protein (<5 mg/L)	88	120	189
Type of patients with COVID-19 (severe/non-severe)	Severe	Severe	Severe
Risk factors	Hypertension	Type 2 diabetes, hypertension	Hypertension, type 1 diabetes, high cholesterol,
The time between the onset of COVID-19 infection and onset of ICH (days)	21	10	12
Location of hematoma	Parietal-occipital region on the left with a breakthrough of blood into the ventricular system	In the projection of the basal ganglia on the right	In the projection of the basal ganglia on the right with a breakthrough into the ventricular system and a median dislocation to the left
Modified Rankin Scale (mRS, 0–6) (discharge)	4	2	5

## Case Presentation

### Patient 1

**FIGURE 1:**
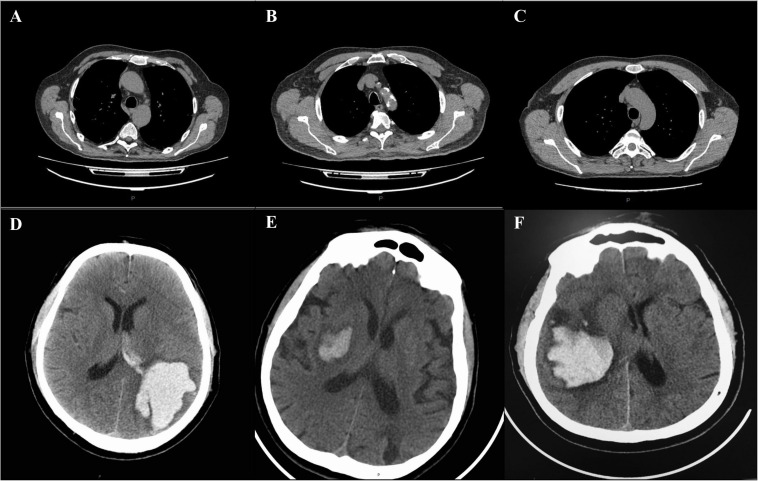
CT changes of lung and brain in patients with COVID-19 complicated with ICH **(A–F)**. Chest and brain CT examination of patient 1 showing bilateral consolidations and ground-glass opacities of the lungs **(A)**, and hemorrhage in the parietal- occipital region on the left with a breakthrough of blood into the ventricular system **(D)**. Chest and brain CT examination of patient 2, showing diffuse bilateral ground-glass opacities involving both lungs **(B)** and hemorrhage in the projection of the basal ganglia on the right **(E)**. Chest and brain CT examination of patient 3, demonstrating bilateral ground-glass opacities **(C)** and hemorrhage in the projection of the basal ganglia on the right with a breakthrough into the ventricular system **(F)**. P = posterior.

A 50- year-old male with well-controlled hypertension presented with a 2-week history of cough, fever and fatigue. A CT chest study demonstrated bilateral ground-glass opacities consistent with COVID-19 pneumonia (Figure 1A). One week post-admission, he became drowsy with new severe headache, right-sided hemiplegia, and right-sided hemihypesthesia. A CT head examination demonstrated an ICH in the parietal-occipital region on the left with a breakthrough of blood into the ventricular system requiring craniotomy and evacuation (Figure 1D). An intracranial CT angiogram (CT-A) was normal. At the time of ICH detection, INR, APTT, platelets and fibrinogen were normal, and the patient was receiving a prophylactic dose of low molecular weight heparin (LMWH). After a 3-week admission, patient was discharged to a rehabilitation center for further therapy.

### Patient 2

A 64-year-old male was hospitalized for COVID-19 pneumonia (7 days history of cough, fever and fatigue) treatment. A CT chest showed typical COVID-19 pneumonia changes (Figure 1B). He had a history of multiple, hypertension and type 2 diabetes mellitus. On day three of hospitalization, he developed left hemiparesis. He had a GCS score of 13 and a blood pressure of 210/90. CT of the brain showed ICH in the projection of the basal ganglia on the right, which did not require surgical intervention (Figure 1E). CT-A of the brain was normal. The patient was admitted to the hyper-acute stroke unit (HASU) for further care. After a 2-week admission, he was discharged to a rehabilitation center for further therapy.

### Patients 3

A 60-year-old male with a history of hypertension, type 1 diabetes mellitus and hyperlipidemia, presented with a 5 days history of shortness of breath, cough, fevers, and pleuritic chest pain. A CT chest study demonstrated bilateral ground-glass opacities consistent with COVID-19 pneumonia (Figure 1C). Clinical and laboratory evaluation showed moderate respiratory distress (PaO2/FiO2 190). The respiratory function progressively worsened during the following days and, on day six, he was transferred to an intensive care unit (ICU) for invasive ventilation. The next day, the patient was found with bilaterally fixed and dilated pupils, with a GCS of 3. Brain CT examination showed a new ICH in the projection of the basal ganglia on the right with a breakthrough into the ventricular system requiring craniotomy and evacuation (Figure 1F). The ventricles were displaced across the midline. CT-A of the brain was normal. Coagulation tests were normal. A total of 6 weeks post-admission the patient remains on ICU, receiving multiple organ support.

## Discussion

We report three cases with severe COVID-19 infection who developed an ICH. Therefore, the question is whether COVID-19 infection is the cause of ICH, or is it a coincidence with ICH. All patients had risk factors for ICH like hypertension and DM. During the penetration of COVID-19 into the cells of the body, angiotensin-converting enzyme 2 (ACE2) plays a crucial role (Weimar and Kleine-Borgmann, 2017). Through ACE2, not only does the viral infection penetrate into the cell, but with COVID-19, the expression of ACE2 decreases, which leads to dysfunction of the renin-angiotensin-aldosterone system (RAAS) and damage to the lungs and other organs and systems (Weimar and Kleine-Borgmann, 2017). A decrease in ACE2 expression can increase the risk of ICH in several ways: (1) a decrease in ACE2 expression can increase local Angiotensin II (Ang II) levels, which, acting on AT1 receptors, can increase blood pressure; (2) ACE2 deficiency in the CNS can lead to endothelial dysfunction in the cerebral vessels, leading to an increase in the risk of a cerebral hemorrhage; (3) A decrease in ACE2 expression will also lead to a decrease in Ang (1–7) generation and depression of Ang (1–7)/MasR signaling, thereby preventing its vasodilatory, neuroprotective, and antifibrotic effects (Weimar and Kleine-Borgmann, 2017; Benger et al., 2020; South et al., 2020). Therefore, it is reasonable to conclude that COVID-19 may exacerbate hypertension and increase the risk of ICH in patients.

Diabetes mellitus is also an independent risk factor for the development of ICH. Several biological mechanisms could explain the observed association between high glucose and ICH. High glucose could impair normal endothelial function, and subsequently lead to brain small vessel disease (SVD). Degenerative changes in the walls of brain small vessels could cause ICH (Boulanger et al., 2016). Moreover, the rate of ICH in DM patients with the hypertension is higher than those without hypertension (Benger et al., 2020). Both hyperglycemia and hypertension can induce the risk factors to act on the brain vessels and to make them easy to be ruptured. DM is a chronic inflammatory condition characterized by multiple metabolic and vascular disorders that can influence our response to infection pathogens, in particular COVID-19 (Boulanger et al., 2016). Hyperglycemia and insulin resistance increase the synthesis of proinflammatory cytokines, oxidative stress, and stimulate the production of adhesion molecules that mediate tissue inflammation (Tadic et al., 2020). This inflammatory process may be one of the main mechanisms that lead to a higher propensity for COVID-19, with worse consequences in DM patients (Tadic et al., 2020). Indeed, the available data indicate that DM ranks second after hypertension among confirmed COVID-19 cases with major chronic diseases (Tadic et al., 2020).

Additionally, all of our patients had elevated D-dimer and C-reactive protein (CRP) on admission in the setting of COVID-19. The most common pattern of coagulopathy observed in patients hospitalized with COVID-19 infection is characterized by elevations in fibrinogen and D-dimer levels (Becker, 2020). In fact, patients with severe COVID-19 were reported to have increased D-dimer and tissue plasminogen activator (tPA) plasma levels, both of which are associated with an increased propensity for hemorrhagic complications (Becker, 2020; Divani et al., 2020). Therefore, it is possible that a coagulopathy in the setting of activation of intrinsic and extrinsic fibrinolytic processes predisposed these patients to ICH. The serum inflammatory factors, such as CRP play a great role in the process of the vascular damage (Boulanger et al., 2016). Early stage COVID-19 CRP levels are known to positively correlate with lung involvement and may reflect disease severity (Wang, 2020). Some researchers found that the incidence of ICH was significantly higher in the high CRP group than in the low CRP group (Liu et al., 2014). It indicates that CRP may influence the incidence of ICH in COVID-19 patients.

## Conclusion

While it remains to be confirmed whether there is a causal relationship between COVID-19 infection and ICH, these three cases, along with others in the literature, support that COVID-19 patients with severe illness are at risk for ICH.

## Data Availability Statement

All datasets presented in this study are included in the article/supplementary material.

## Ethics Statement

The studies involving human participants were reviewed and approved by the Ethics Committee of the Hospital COVID-19 Clinics BSMU in Ufa, Russia (within the Department of Neurosurgery), Clinic of the Bashkir State Medical University, Ufa, Republic of Bashkortostan, Russia, and Bashkir State Medical University. The patients/participants provided their written informed consent to participate in this study. Written informed consent was obtained from the individual(s) for the publication of any potentially identifiable images or data included in this article.

## Author Contributions

VP contributed to conception and organization of research project, drafted the main manuscript text. OB and IG performed the experiments and acquired the data. LT and AS were involved in study conception, participated in design and coordination, and helped to draft the manuscript. GA was responsible for data acquisition, writing – review and editing, analysis and interpretation of data, wrote the manuscript, and approved the final version of the manuscript. All authors have read and agreed to the published version of the manuscript.

## Conflict of Interest

GA employed by GALLY International Biomedical Research LLC. All of other authors declared no conflict of interest.

## Footnotes

1 http://ethics.iit.edu/ecodes/node/3931
